# Guidance and absorption of internal energy in Vivaldi antennas using multiple slots, full-band dividers, metamaterials, and distributed resistors

**DOI:** 10.1038/s41598-026-39126-x

**Published:** 2026-02-21

**Authors:** Ha Hoang, Minh-Huy Nguyen, Vinh Pham-Xuan

**Affiliations:** 1 Department of Telecommunications Engineering, Ho Chi Minh City University of Technology (HCMUT), VNU-HCM, Ho Chi Minh City, 700000 Vietnam; 2 Dassault Systèmes Deutschland GmbH, Darmstadt, 64289 Germany

**Keywords:** Engineering, Electrical and electronic engineering

## Abstract

In traveling-wave planar-technology antennas, only a certain portion of excitation energy contributes to expected radiation. The remaining significant portion, referred to as residual electromagnetic (EM) energy, has negative effects on overall antenna performance. A systematic design methodology to mitigate such degradation is presented in this paper. The design approach is based on an analysis of structural scattering and radiation mechanism, supported by near-field and partition far-field characterizations. The design process involves the arrangement of primary antenna scattering sections into a form of multi antipodal Vivaldi slots; the identification of desired and undesired EM flows of internal EM energy; the adjustment of radiation flows using inhomogeneous metamaterial structures; and the guidance and absorption of residual energy through dissipation paths with distributed resistors. The effectiveness of the method is demonstrated by the high-gain and wideband antenna performance. Additionally, novel full-band power dividers are proposed to efficiently feed both individual antenna and array.

## Introduction

Functional and performance requirements of a radiation subsystem - antenna in modern wireless systems, which is used for communication, remote sensing, harvesting or a combination of these purposes, are becoming increasingly diverse and demanding. In addition to primary functions of wireless transmission and reception, the capability of an antenna to dynamically alter its radiation features to meet specific operational requirements are essential in the next generation systems. This capability encompasses features such as beamforming, full-duplex operation, and the reconfigurability of operating modes, frequency and polarization^1–11^. The performances of antennas are being progressively driven toward their theoretical limit for maximum benefits. For instance, achieving high directivity across a broad bandwidth for an antenna with size constraints represents one of the most critical design challenges. This, in turn, maximizes the system efficiency of energy transfer across the operational bandwidth and minimizes EM energy occupation in the propagating space and time domains.

Traveling-wave structures are capable of supporting both high directivity and wideband performances, with Vivaldi antenna being a prominent example that can be realized using planar technology. Significant research efforts have been dedicated to improve these performance metrics of Vivaldi antenna. The common approach is to modify the original geometry of the Vivaldi antenna, such as customizing the Vivaldi wings shape into the alternative shapes, e.g. elliptical, circular or leaf shapes^12–14^, to extend the propagation paths for internal EM energy flows, thereby improving gain and bandwidth. Similar improvements were achieved using etching peculiar shapes on the wings^15,16^. Additionally, secondary order EM scattering flows were redirected toward the main radiation direction by incorporating etching slots^17–20^ into the wings. Further gain enhancement was achieved by introducing a core into the Vivaldi slot or designing a double slot structure^21^. Zero-index metamaterial (ZIM) perspective was proposed in^22^ where the zero-index property equivalence hypothesis of the metamaterial was used to explain the flattening of wavefronts under the influence of the metamaterial at the antenna radiating apertures within the zero-index frequency bands^23,24^.

In conventional design methodologies, the lack of near-field characterization on antenna structure hinders the effective optimization of antenna overall performance. To address this issue, near-field response analyses and characterizations have been introduced in^25–27^ to gain insights into the interactions between EM fields and structural sections of the antenna. The analyses provide more rigorous understanding of the propagation and radiation mechanisms within structural sections of Vivaldi antennas. Additionally, the characteristics assist a systematic evaluation of the impact of local near-field response at each structural section on the overall antenna performance. This allows the identification of key factors and optimization strategies for individual structural sections to effectively achieve an optimized overall antenna response.

In this work, a novel approach in analysis and design of a high gain and wideband traveling-wave planar antenna is proposed. The methodology using near-field response analysis is applied to design antipodal Vivaldi-slot-based structures. The near-field and partition far-field characteristics^27^ help to identify the key structural sections involved in the propagation process, and importantly to classify internal EM energy into different EM energy flows. The impacts of the desired and undesired EM flows on the overall antenna responses are evaluated. Additionally, various phenomena in the propagation and radiation process within the structures are physically interpreted. Based on these analyses and considering the structural geometry, the multi-slot antipodal Vivaldi structure emerges as an optimal solution within planar technology, achieving both wideband and high-gain performance. Moreover, the propagating conditions of the scattering EM flows within the Vivaldi slots are analyzed and optimized through the adaptive integration of inhomogeneous metamaterial structures into the Vivaldi slots. This leads to an improvement of the propagation conditions, thereby contributing to a substantial enhancement of the overall antenna performance.

A classification of internal EM energy flows into desired and undesired/residual components enables an analysis of their impacts on overall antenna performance. This analysis reveals performance degradation attributed to residual EM energy flows and identifies the structural sections where residual energy is concentrated. This information aids the design of dissipation paths that guide residual EM energy toward distributed resistors for effective absorption. The absorption of residual EM energy is optimized using different strategies to achieve specific performance objectives for the overall antenna response. The optimizations effectively mitigate the negative impacts of residual EM flows, resulting in a significant enhancement of the antenna performance.

The implementation of the feeding for the multi-slot structure and an array consisting of four such antennas is realized using novel power dividers. The design of these dividers is based on a combination of serial/parallel T-junctions and single/multi-level hierarchies to construct novel full-band, uniform-port-impedance $4^n$-port dividers. The performance of these dividers is presented through their ability to deliver power with high-efficiency and maintaining both magnitude and phase balance across all output ports in full-band. This approach ensures the preservation of the desired responses of both the individual antenna and the array over the entire bandwidth.

The subsequent sections of the article are organized as follows: Section Multi-slot Vivaldi structures and field features presents an analysis of field features and explains the antipodal Vivaldi multi-slot structuring for antennas. Section Scattering flow adjustment with metamaterial introduces the inhomogeneous metamaterial structures and demonstrates its effectiveness in adjusting propagation condition. Establishment of the dissipation paths with distributed resistors for residual energy absorption and absorption optimization are presented in Section Residual energy guidance and absorption. Novel full-band feeding structures are presented in Section Realization of multi V-slot and array antenna with novel full-band power dividers to realize the feeding for the proposed antenna and array. Finally, Section Conclusion concludes the paper.

## Multi-slot Vivaldi structures and field features

Within planar antenna technology, primary scattering sections of the antipodal Vivaldi structure are directly connected to the excitation port. As a result, the radiation bandwidth of the antenna is not influenced by excitation couplings, stubs, baluns or transformers. Consequently, the antipodal Vivaldi emerges as a strong candidate for full-band or wideband applications, and serves as a fundamental radiating element in the design proposed in this study.

**Fig. 1:**
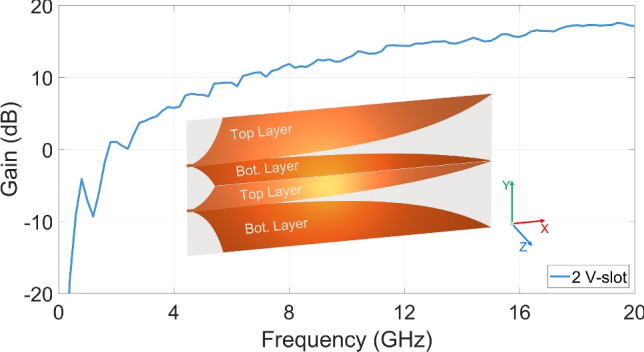
2 V-slot antenna structure and gain.

In this work, the antenna structures were designed using Roger RT5880 substrate with a dielectric thickness of $0.254 ~ \text {mm}$ and a metal layers thickness of $35~\mu \text {m}$. The $50 \ \Omega$ impedance excitation strip line for an antipodal Vivaldi slot has a width of $0.97~ \text {mm}$. The analyses were conducted over a frequency range of 0 to 20 GHz.

The first two-slot antipodal Vivaldi, referred to as the 2 V-slot, was designed with a dimension of $132 \times 60~ \text {mm}^2$ and ports spacing of $20~ \text {mm}$. To preserve the antipodal characteristics for the two Vivaldi slots and ensure the electrical isolation between the two wings at the center, the four metallic wings were arranged across the two metal layers in a top layer – bottom layer – top layer – bottom layer (T-B-T-B) sequence, as illustrated in Fig. 1. The two ports were excited to create a balance field in the two Vivaldi slots. The realized gain in the main direction exhibits an increasing trend with frequency. While the gain reaches approximate 17 dBi at 19 GHz, there are significant ripples over the operable bandwidth from 2 to 20 GHz. Understanding the cause of these ripples and mitigating this effect remains challenges to be addressed.

**Fig. 2:**
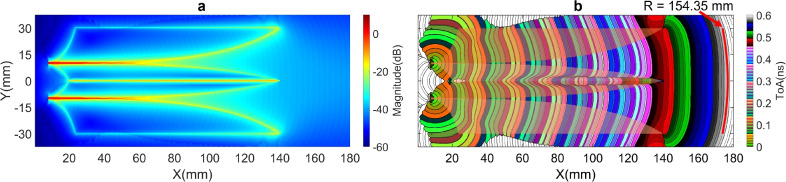
(**a**) Magnitude and (**b**) ToAs of the first peak Poynting clusters in 2 V-slot structure.

The visualization analysis of near-field energy cluster distributions on the antenna structure in time domain^25–27^ enables the identification of the dominant EM energy flows along the paths. The energy intensity and time of arrivals (ToAs) of the first clusters of the Poynting vector were evaluated on the middle substrate layer of the antenna. As shown in Fig. 2a, the Vivaldi edge regions of the four wings are the primary propagation paths for the EM energy, guiding the EM flows from the excitation sources to generate first order scattering EM flows within the two Vivaldi slots. These energy flows contribute significantly to the expected radiation energy in the antenna main radiation lobe. Additionally, other edge and patch areas on the metal wings also guide a significant portion of EM energy flows generating first- and higher-order scattering EM flows. Some of these EM energy flows might propagate along the primary paths but in the directions opposite to the source flows. Due to differences or non-correlations in the geometry of these paths; and the propagation directions and time responses of undesired EM flows in these paths, compared to the source flows in the primary paths, their resulted scattering energy does not improve the expected radiation but rather degrades it, particularly in wideband operation and under resonant effect over extended analysis period. This phenomenon explains for the ripple observed in the antenna gain as shown in Fig. 1. In this work, an EM energy flow, propagating along any non-primary paths, as well as any flow, propagating along the primary paths but in the opposite direction or out of the first cluster ToA period, are defined as residual EM energy flows.

**Fig. 3:**
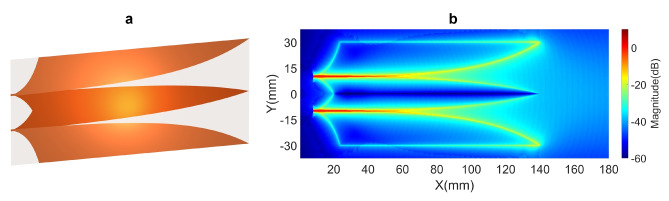
(**a**) 2 V-slot structure with connected core wings and (**b**) near-field intensity characteristic.

In some cases, residual EM flows can be leveraged to improve antenna performance. An example is the 2 V-slot structure shown in Fig. 3. In contrast to the structure in Fig. 1, the two core wings at the center are on the same metal layer and connected together. This core structure allows some EM energy flows from one Vivaldi core edge to propagate across the core to the opposite Vivaldi core edge, thereby generating additional scattering EM energy into the Vivaldi slot. Thus, the total radiation at the antenna aperture is increased, leading to an improved gain. However, the radiation bandwidth is limited due to the difference in propagating path lengths between the source flow and crossing-core flows as mentioned above. Furthermore, since the two core wings are directly connected, the method for absorbing residual EM energy flows, introduced in section Residual energy guidance and absorption, is not applicable for this structure. Consequently, this structure was not investigated further in this study.

The ToAs contour lines in Fig. 2b represents wavefronts. At the antenna aperture, the flatness of these wavefronts is corresponding to the parallelism and co-phase/ToA of radiation flows and this flatness is proportional to antenna directivity. The flatness is measured by a fitting circular arc with a radius of R, which is used as a near-field characteristic to evaluate antenna directivity. As illustrated in Fig. 2b, the measured R radius for the 2 V-slot antipodal structure is $154.35 ~ \text {mm}$.

**Fig. 4:**
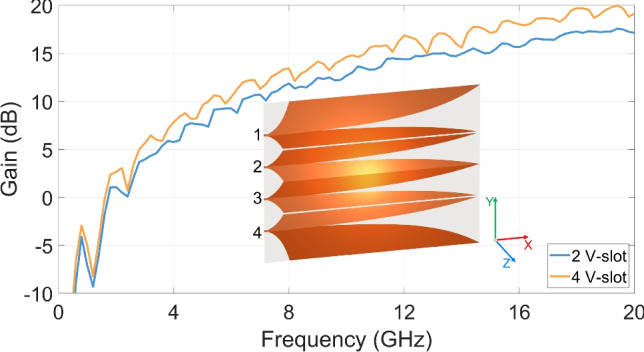
4 V-Slot structure and gain.

The 2 V-slot antipodal structure depicted in Fig. 1 has been extended to a 4 V-slot antipodal antenna as illustrated in Fig. 4. By maintaining the port spacing and V-slot length, antenna size only increases in the Y-direction and reaches a total size of $132 \times 100 ~ \text {mm}^2$. To preserve the antipodal characteristics for the 4 V-slots and to ensure the compatibility with excitation using a power divider, the 8 wings were arranged across the two metal layers in the sequence T-B-B-T-B-T-T-B. In this structure, in contrast to the electrical isolation of the wing pair of the center core, each wing pair of the upper and lower cores is connected together. Two 1 mm isolation gaps were introduced in the centers of these cores to achieve electrical isolation for all wings, as illustrated in the Fig. 4.

The four ports were uniformly excited to establish a balance field distribution across all 4 V-slots. As the radiation aperture width is doubled, the realized gain of the 4 V-slot structure is increased approximately 3 dB at the highest scattering efficiency frequencies. However, as shown in Fig. 4, the amplitude of the ripples in gain increases significantly when compared to the 2 V-slot.

Consideration of the influence of the desired and residual EM energy flows provides insights into the increases in the gain as well as the ripple amplitude observed in the 4 V-slot response as compared to the 2 V-slot. Assuming that the responses to the balance source EM flows along the primary paths of all the V-slots are almost uniform. Doubling the number of V-slots from 2 to 4, the number of excitation sources and total excitation power are doubled. The superposition of the main radiating field from twice as many V-slots forms a far-field, whose amplitude and power density are doubled and quadrupled, respectively. When the excitation power of the 4 V-slot is normalized with the 2 V-slot, the 4 V-slot far-field power density remains twice that of the 2 V-slot. This alternatively explains for the approximate 3 dB gain increase of the 4 V-slot compared to the 2 V-slot, where 3 dB is also the maximum gain increase by doubling the number of uniform slots. This also demonstrates the optimized effectiveness of the multi-V-slot structuring when V-slot size is minimized.

Due to the significant near-field influence between adjacent wings, the responses to residual EM energy flows in the core and lateral wings are significantly different. This difference is also enhanced by structural difference of the two types of these wings. In the 2 V-slot structure, the ratio of core wing number to lateral wing number is 2:2, while this ratio is 6:2 in the 4 V-slot structure. The increase in a number of the similar wings enhances the correlation in response to similar residual EM flows in the wings. This magnifies negative impacts of the residual EM flows to superposition far-field in the main radiating direction, and results in the increase of ripple amplitude in the gain of the 4 V-slot.

**Fig. 5:**
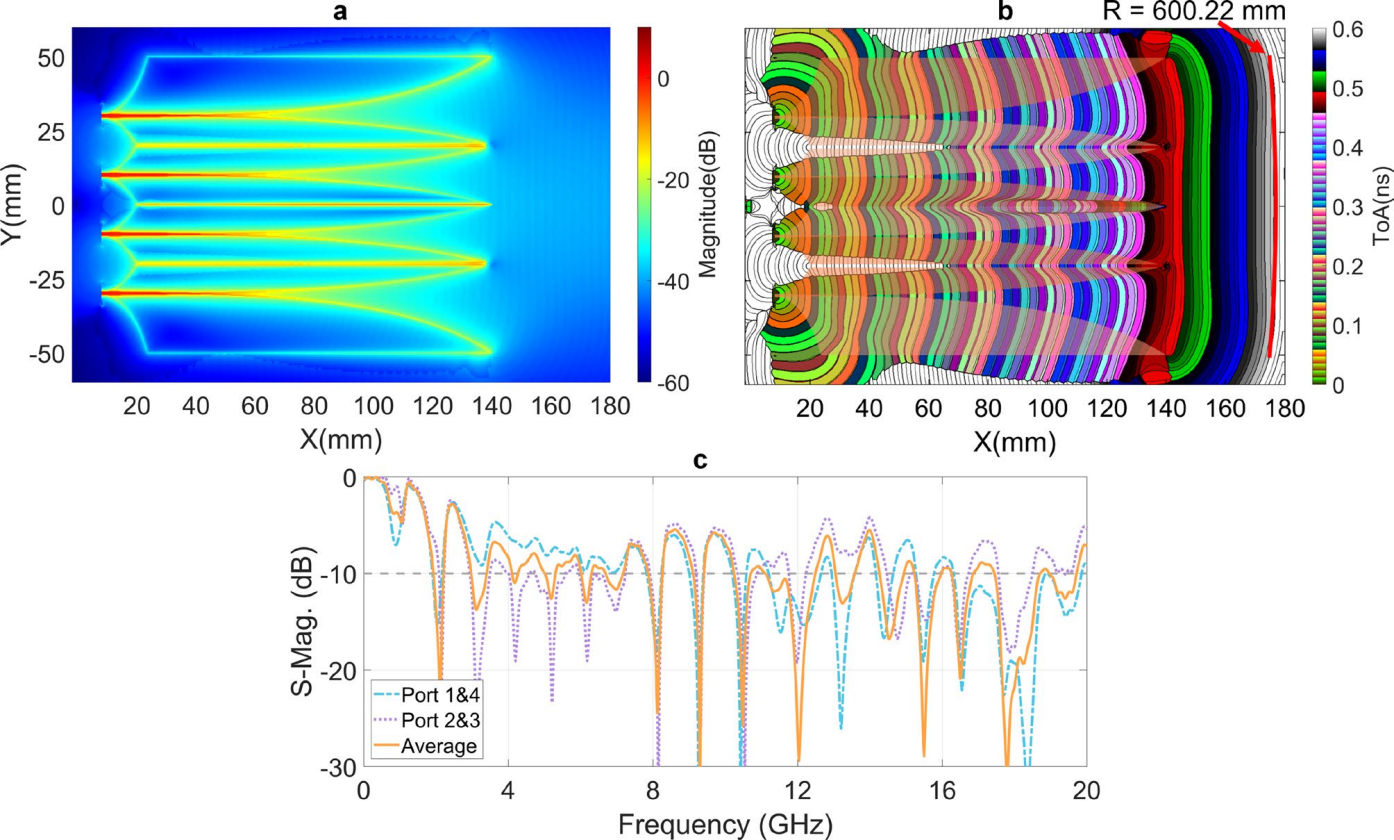
Near-field characteristics of 4 V-slot structure.

Figure 5 shows the near-field characteristics of the 4 V-slot antenna under the simultaneous and balanced excitation at the four ports. The responses to the balance source EM flows along the primary paths of all the V-slots are almost uniform as shown in Fig. 5a. The intensity of the scattering fields is also distributed relatively uniform across the 4 V-slots with only a slight reduction observed at the two outer edges of the antenna aperture ($\text {X} > 140 ~ \text {mm}$), corresponding to the two lateral wings. Across the three cores, the field responses of the core wings exhibit no significant difference, despite the fact that the two wings at the center are located on different metal layers, while the wing pairs in the upper and lower cores are on the same metal layers. Figure 5b presents a significant improvement in the wavefront flatness, with the wavefront radius R reaching $600.22 ~ \text {mm}$. This near-field enhancement results in an increase of approximately 3 dB in far-field gain for the 4 V-slot, as compared to the 2 V-slot.

Figure 5c shows the S-parameters at the four excitation ports. Due to the balance of the structure and the excitation, port 1 and port 4 exhibit similar responses, as do port 2 and port 3. The average S-parameter across all four ports is also calculated and shown in the figure. Significant residual EM flows along non-Vivaldi edges of the wings are observed in Fig. 5a. While a portion of this residual EM energy negatively impacts the radiation by causing the ripples in gain, another portion considerably reflects back into the excitation ports, as evidenced by the S-parameter results in Fig. 5c.

Near-field characterization enables the decomposition of internal EM energy into distinct EM flow components. The multi V-slot structure functions as an effective structural support for excitation EM flows, generating scattering flows with more flattened wavefronts at the antenna aperture. However, under the constraints imposed by the planar technology, size and frequency band, the excitation EM energy flows cannot be completely radiated within the expected timeframe. The remaining EM energy retained within the structure is referred to as residual EM energy flows, which negatively impact the desired radiation performance. The methods introduced in the subsequent sections employ novel strategies to progressively improve the desired scattering characteristics and mitigate the negative effects of these residual flows.

## Scattering flow adjustment with metamaterial

The effectiveness of metamaterials in changing propagation conditions has been demonstrated in previous researches. In particular, the application of metamaterials to Vivaldi antennas operating within the zero-index band has been discussed in^22–24^ to significantly improve the antenna performance by effectively flattening the wavefronts at the antenna aperture. This phenomenon was explained using the hypothesis of the equivalence in field response between the metamaterial and the homogeneous material with zero-index property. In this section, the metamaterials are applied to the 4 V-slot antenna to enhance the radiated response.

**Fig. 6:**
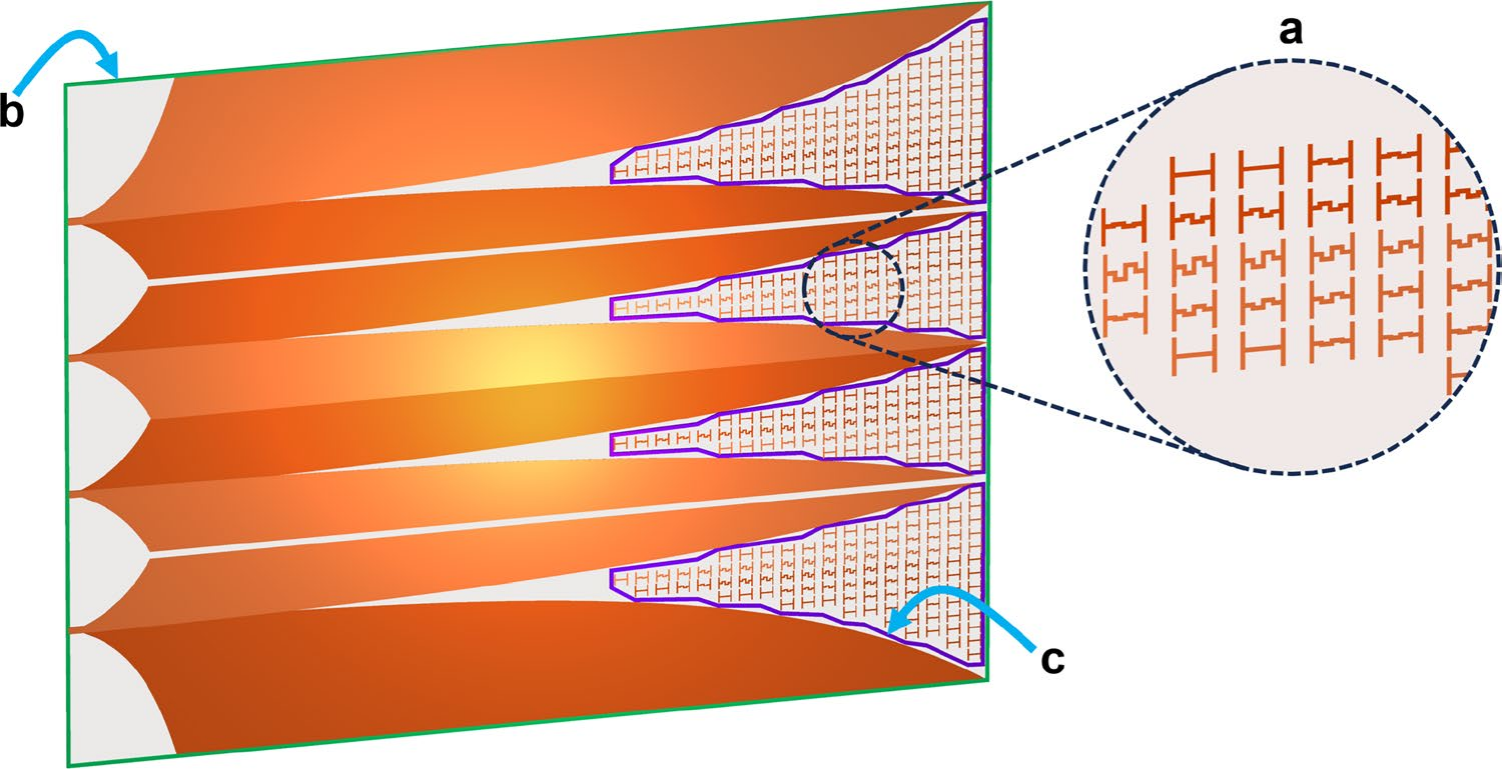
(**a**) Distribution of inhomogeneous metamaterial I-elements on V-slots and (**b**, **c**) boundaries used for partition far-field responses calculation.

**Fig. 7:**
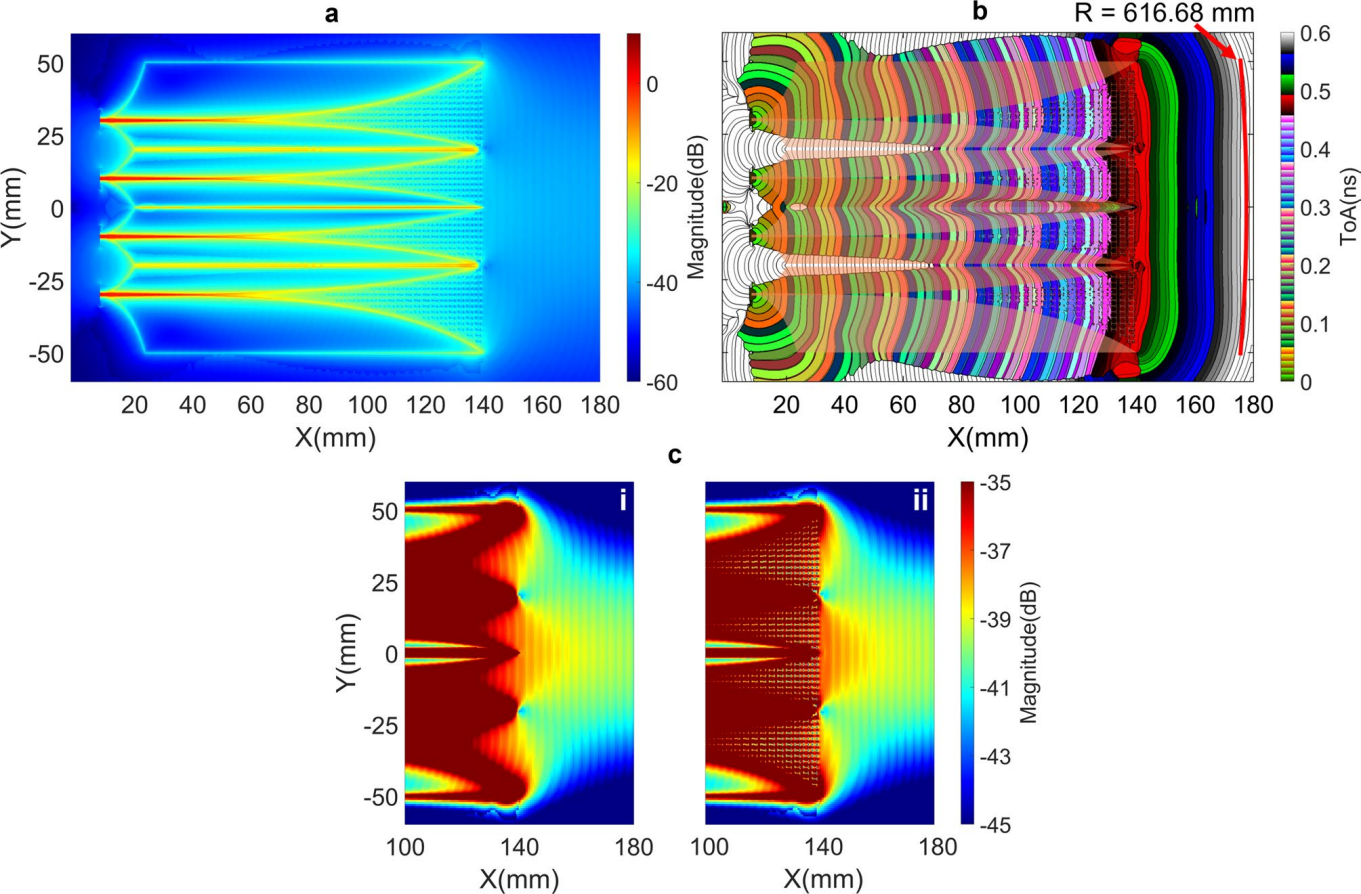
(**a**, **b**) Near-Field characteristics of 4 V-slot with metamaterial and (**c**) comparison of near-field intensities on the structures (c-i) without and (c-ii) with metamaterial.

The curvature of the ToAs contour lines in each V-slot in the regions $100 ~ \text {mm}< \text {X} < 140 ~ \text {mm}$, depicted in Fig. 5b, indicates that the scattering EM energy flows in each V-slot is not in-phase. Consequently, the superposition of these EM flows is suboptimal to form the flat wavefronts at the radiating aperture $\text {X} > 140 ~ \text {mm}$. To enhance wavefront flattening in the V-slots, propagation condition for the scattering EM flows was changed by adaptively integrating inhomogeneous metamaterial structure into each V-slot, as illustrated in Fig. 6a. Each element of the metamaterial structures has dimensions of $1.96 \times 1.7 ~ \text {mm}^2$ and has either a straight or meandered I-shape. The length of the meandered line in each I-element varies depending on its position within the V-slot. The propagation delay of an EM flow along an I-element in the X-direction increases with the length of the meandered line.

Metamaterial I-elements are spaced uniformly along the X-direction to fill the four V-slots. As a result, the number of elements along the X-direction is greater near the center of the V-slot, as compared to regions closer to the Vivaldi edges. This difference in number of elements causes EM energy propagating through the center of the V-slot to experience greater delay, thereby slowing the EM flows at the center more than those near the edges, contributing to the wavefronts flattening. Additionally, I-elements with different meandered line lengths, as illustrated in Fig. 6a, were strategically positioned within the V-slot to adjust the EM flow speed more accurately at detail areas in each V-slot. As a consequence, the wavefronts within each V-slot are more effectively flattened.

Each antipodal V-slot in the antenna, as shown in Fig. 6, comprises two wings fabricated on separate metal layers located on opposite sides of the substrate. Accordingly, each half of the metamaterial structure within a V-slot was placed on the side corresponding to its respective wing, as illustrated in Fig. 6a, to conform to the Vivaldi edge profile of each wing. The distinction between the top and bottom layers is indicated by color, with the lighter shade representing the top layer and the darker shade representing the bottom. This arrangement ensures the establishment of proximity coupling between the I-elements and the Vivaldi edge, enhancing the scattering efficiency of the EM flows along the edges.

Figure 6 also defines the area boundaries used for the evaluation of contributions of specific volumes within the antenna structure to the far-field response, following the method introduced in^27^. Figure 6b outlines the area of the entire antenna structure, while Fig. 6c illustrates the region encompassing a single metamaterial structure within one V-slot. The partition far-field response characteristics associated with these bounded volumes include partition gain in frequency domain and far-field time response. These quantities represent the contribution of the local sections of the structure to the overall radiation of the antenna.

The intensity and ToA distributions of the first clusters of Poynting vectors for the antenna with metamaterial structures are depicted in Fig. 7a and 7b. A comparison of the intensity at the antenna aperture, without and with the metamaterial structures, is presented in Fig. 7c-i and 7c-ii, which clearly demonstrates an enhancement in intensity when the I-elements are integrated to the V-slots. The improvement in wavefront flattening within each V-slot is evident when comparing the ToAs contour lines within each V-slot in the regions $100 ~ \text {mm}< \text {X} < 140 ~ \text {mm}$ between Fig. 5b and Fig. 7b. This also contributes to the flattening of the overall radiating wavefronts at the antenna radiating aperture, as reflected by the increase in the matched circle radius from $600.22 ~ \text {mm}$ in Fig. 5b to $616.68 ~ \text {mm}$ in Fig. 7b.

**Fig. 8:**
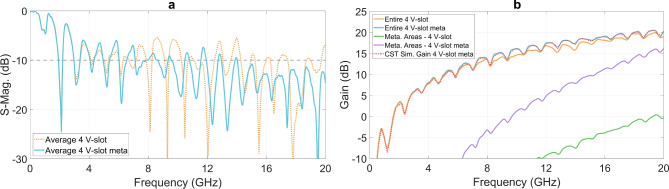
The effectiveness of the metamaterial on (**a**) the average of S-parameters of four ports and (**b**) partition gains.

The positive impact of the metamaterial structure on S-parameter is presented in Fig. 8a. The reduction in EM energy reflection back to the ports within the 7 to 20 GHz band, as shown in this figure, indicates an improvement in EM scattering.

Far-field responses in the main radiating direction of the entire structure and a partitioned section, are presented in Fig. 8b. The gain of the entire structure, comprising 4 V-slots integrated with the metamaterial I-elements, exhibits an improvement of approximately 1 dB within 7 to 20 GHz band, as compared to the structure without the metamaterial. The evaluation of the entire structure gain was further validated through comparison with simulation result obtained using SIMULIA CST Studio Suite^®^^28^, demonstrating a good agreement, as shown in Fig. 8b. To illustrate the increased antenna gain due to the addition of metamaterials, the partition gain contributions from the EM near-field regions, delimited by four boundaries (c) in Fig. 6, are presented in Fig. 8b. The partition gain from the areas filled with metamaterials (purple curve in Fig. 8b) is significantly higher than that of the areas without metamaterials filling (green curve). This clearly demonstrates that adding metamaterials contributes to an improvement in antenna gain in the main radiation direction.

The integration of the metamaterial into the antenna structure further facilitates the scattering of the desired EM energy flows and controls the scattering EM flows within the 4 V-slots to improve intensity and flatness of wavefronts at the antenna aperture. However, the negative effects of residual EM energy flows are still present. These include high near-field intensity at the lateral wing edges and isolation core gaps, as observed in Fig. 7a. This leads to high reflection of energy back to the ports at lower frequencies, as shown in Fig. 8a, and gain ripples in Fig. 8b. These issues will be addressed in the next section.

## Residual energy guidance and absorption

Constraints related to geometrical shape, dimensions and material composition of the main propagation paths prevent the excited dynamic EM energy within the desired frequency band to be completely radiated within the expected timeframe. The remaining EM energy retained within the structure, referred to as residual EM energy, considerably degrades antenna performance.

In this section, a novel approach is proposed to mitigate the residual energy by establishing dissipation paths with integrated resistors. These dissipation paths attract and guide the residual energy toward resistors mounted at appropriate positions on the paths to absorb the undesired energy. Values of these distributed resistors can be optimized using different strategies to achieve specific objectives of the overall antenna response.

### Establishment of dissipation paths with integrated distributed resistors

A lateral wing is on the antenna side, and its lateral edge is not adjacent to any other wing. It is a floating electrode to its excitation source at DC. The main path for expected radiating is the area along Vivaldi edge of the wing, and it facilitates the scattering of the excitation EM flow generating the main radiation lobe. Other areas of the lateral wing were considered to guide undesired EM flows.

**Fig. 9:**
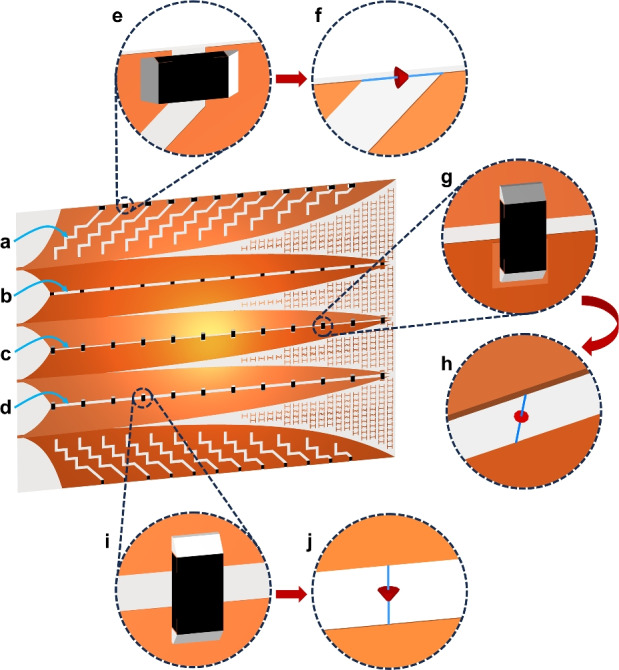
Establishment of dissipation paths using (**a**) zigzag slots on lateral wings, (**b**, **c**, **d**) isolation gaps for cores; with (**e**, **g**, **i**) mounted distributed resistors on slots/gaps and (**f**, **h**, **j**) simulation models.

**Fig. 10:**
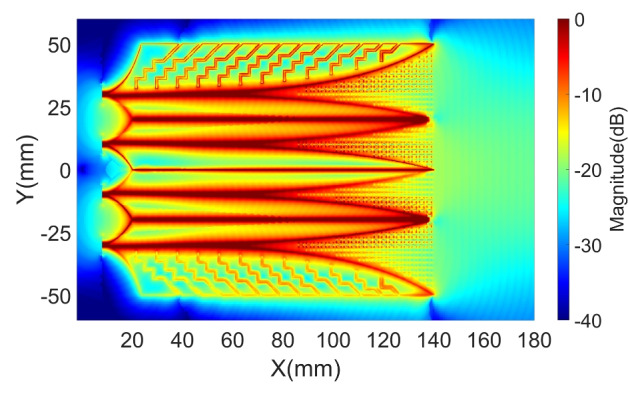
Concentration of EM energy on zigzag slots.

Due to the continuous distribution and indetermination of the residual EM energy flows, they must be attracted and guided in the specific paths toward the discrete resistors for effective absorption. This was achieved by using twelve zigzag slots, referred to as dissipation paths, as shown in Fig. 9a. These slots are adaptively etched onto each lateral wing, starting at the point 2.5 mm from the Vivaldi edge and terminating at the lateral edge of the wing. At each zigzag slot, potential and current differences are induced on opposing sides due to differences of the initial residual EM flows and disturbances in the residual EM energy on two metallic patches connected to both sides of the slot. With emergence of potential and current differences on the two slot edges, each slot functions as an efficient mechanism to capture residual EM energy from both sides and guide this energy along its length. The zigzag geometry of the slots, together with their 45-degree angular orientation relative to the X-axis, increases the length of the dissipation paths. This extended path length amplifies the potential differences across the slot, thereby attracting more residual EM energy, particularly in the lower frequency band. Concentration of residual EM energy flows along the zigzag slots is demonstrated by the result of the first clusters Poynting intensity on the metal layer of the upper lateral wing as shown in Fig. 10.

**Fig. 11:**
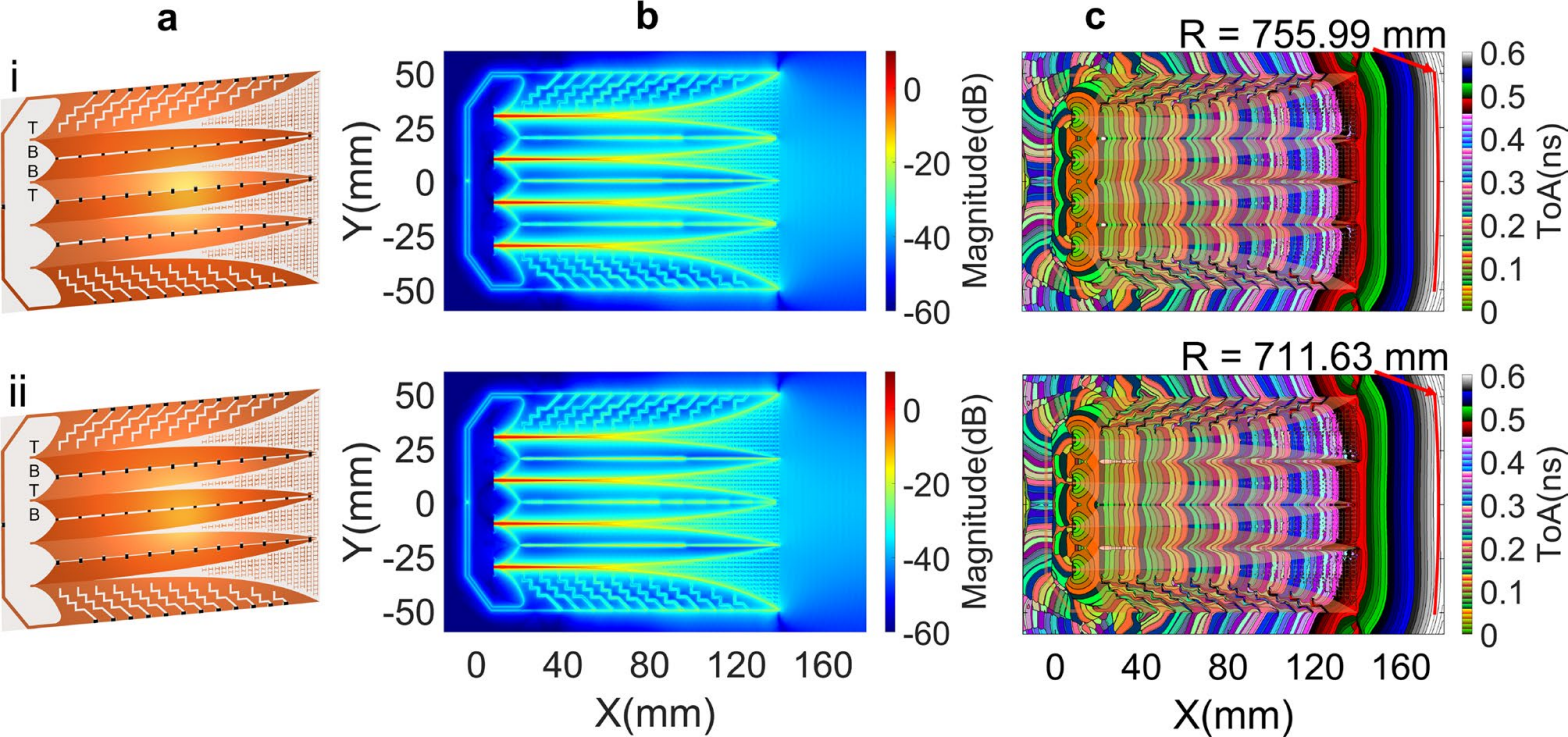
(**a**) Two structures with difference in wings metal-layers arrangement, both employing the same set of optimized distributed resistors and (**b**, **c**) the corresponding near-field responses.

For the side cores, where the two core wings reside on the same metal layer, to isolate the two wings a gap with a width of 1 mm is etched along the centerline of each core, as illustrated in Fig. 9b and 9d. In contrast, an isolation gap is inherently present in the central core, as its two wings are separated by being located on different sides of the substrate, as shown in Fig. 9c. The electric potentials on the two wings of each core are opposite due to the balanced excitations at the four input ports. This potential difference is exploited to attract residual EM energy on the core wings. The field intensity sampled at the mid-plane of the substrate for the structure without zigzag slots as shown in Fig. 7a; and the intensity sampled at the top metal layer for structure with zigzag slots as shown in Fig. 10, both indicate that the residual EM energy is attracted and concentrated along the isolation gaps of the cores.

To efficiently absorb residual EM energy on lateral wings, each zigzag slot was terminated by a resistor, as illustrated in Fig. 9e. This leads to a total of 24 resistors uniformly distributed on the two lateral wings. Similarly, 12 resistors were mounted evenly along each isolation gap in the central and side cores, as shown in Fig. 9g and 9i, respectively. For the side cores, the resistors are placed on the same side as the metallic core wings, as depicted in Fig. 9b and 9d. In contrast, for the central core, where the two wings are positioned on opposite sides of the substrate, the resistors are mounted on the top metal layer and connected to the bottom layer wing via pads and cross-layer interconnects, as shown in Fig. 9c and 9g. In total, 60 resistors are mounted on the entire structure. In simulation, these resistors were modelled as lumped elements, as illustrated in Fig. 9f, 9h, and 9j.

As previously mentioned, the balanced excitation at the input ports results in opposite potentials on adjacent core wings. Consequently, adding resistors across the isolation gaps effectively close the circuit between the two adjacent ports. In contrast, the lateral wings behave as floating electrodes. To close the circuit between the two outer ports, a back loop in series with a resistor was introduced into the structure, as shown in Fig. 11a-i. This addition improves the antenna performance particularly at low frequencies, and increases the total number of resistors in the structure to 61.

### Optimization of residual energy absorption

In the previous subsection, the analysis of the near-field characteristics is crucial in optimizing the design of the dissipation paths and determining the placement of distributed resistors to effectively absorb residual EM energy. The optimization of the values of distributed resistors is presented in this subsection to maximize the absorption of residual EM energy. To accelerate the optimization, two network scattering parameter models were extracted from full-wave simulation models of the two different 4-V slot structures: a 64-port model for the configuration without a back loop and a 65-port model for the configuration with a back loop, as demonstrated in Fig. 9 and Fig. 11a-i, respectively. The 64-port model consists of four antenna excitation ports and 60 distributed resistors, whereas the 65-port model includes one additional resistor at the back loop.

Assuming an *N*-ports network is combined with $N_1$ excitation sources and $N-N_1+1$ distributed resistors, where the *i*th input port of the network is excited by the *i*th source, $i=1,...,N_1$, and each output port from $i=N_1+1,...,N$ is terminated by a distributed resistor $R_i$. The reflected wave at the *j*th port can be expressed as

1 $$\begin{aligned} \begin{aligned} b_j = \sum _{i=1}^{N_1} S_{j,i} a_i + \sum _{i=N_1+1}^{N} S_{j,i} \frac{R_i - R_0}{R_i + R_0} b_i; j = 1, \dots , N \end{aligned} \end{aligned}$$

where, $S_{j,i}$ is an element of the scattering matrix of the network and $a_i$ and $b_j$ represent the incident and reflected wave at port *i* and *j*, respectively. The reference impedance at the output ports is assumed to be identical and is denoted by $R_0$. Equation (1) is solved for each set of the distributed resistors and the reflection coefficient at the *i*th input port, $i=1,...,N_1$, can be written as

2 $$\begin{aligned} \Gamma _i = \frac{b_i}{a_i}. \end{aligned}$$

The power absorbed by the distributed resistor $R_i$ terminated at *i*th output port, $i=N_1+1,...,N$ is computed as

3 $$\begin{aligned} P_i = \frac{1}{2} \left| b_i\right| ^2 \left( 1 - \left| \frac{R_i - R_0}{R_i + R_0} \right| ^2 \right) . \end{aligned}$$

The reflection coefficients at the input ports and/or the absorbed power of the distributed resistors at the output ports are used to formulate the objective in either the time or frequency domain for the optimization of the values sets of the distributed resistors.

In this work, the optimization for the resistor sets includes the following steps: (i) initialization of variables and algorithm configuration; (ii) minimization of the average reflected waves at the input ports over the desired band of 1.6 to 20 GHz; (iii) if the target reflection level was not achieved within the desired band, the minimization is repeated with increased weights applied to the selected frequency bands. Since the minimization was initially not achieved for the sub-band between 1.8 and 3.2 GHz, a higher weighting was assigned to this sub-band during step (iii) of the optimization process.

Tables 1 and 2 present two sets of optimized resistance values for the distributed resistors corresponding to the structures without and with the back loop, respectively. In addition, the tables report the maximum absorption powers for each resistor, together with the frequency at which this maximum occurs. The maximum absorption powers are normalized to the total excitation power and expressed as percentages. Indices of the twelve resistors in each array increase along the X-coordinate. In the tables, the laterals group lists the resistance values, maximum normalized absorption powers and corresponding frequencies for resistors placed on the two lateral wings, whereas the side cores and the center groups present the values for the resistors on the two side cores and the center core, respectively. Table 2 additionally includes the corresponding values for the resistor connected in series with the back loop, listed in the loop group. The maximum absorption power of the resistors in the operating band 1.6 to 20 GHz in the structure without back loop is $6.92\%$ compared to the total excitation power, while that is $3.21\%$ in the structure with back loop.

The near-field responses of the structure using a back loop and the optimized set of distributed resistors, as shown in Fig. 11a-i, demonstrate a significant reduction in the intensity of residual EM fields at the isolation gaps and zigzag slots, as illustrated in first row of Fig. 11b. This reduction effectively improves the flatness of the wavefronts at the antenna radiation aperture, as shown in first row of Fig. 11c, with the radius R of the matched circle increasing to 755.99 mm.

**Table 1:** Optimized resistances of distributed resistors in the structure without back loop and their maximum normalized absorption power at corresponding frequency.

Index		1	2	3	4	5	6	7	8	9	10	11	12
**Laterals**	Res. ($\Omega$)	0	0	278	590	580	5	0	0	6	76	141	109
	Pmax ($\%$)	0	0	1.49	1.25	1.7	0.39	0	0	0.55	2.03	2.55	1.92
	f(Pmax) (GHz)	-	-	13.16	6.92	2.78	4.86	-	-	5.54	1.84	3.24	4.48
**Side** **Cores**	Res. ($\Omega$)	63	283	758	1044	1000	574	0	170	571	704	611	462
	Pmax ($\%$)	6.92	2.87	2	2.23	2.28	2.62	0	1	0.73	0.51	1.01	3.47
	f(Pmax) (GHz)	1.6	1.6	2.06	2.2	2.3	4.68	-	6.58	9.06	12.76	12.74	11.6
**Center**	Res. ($\Omega$)	0	0	441	239	288	588	156	0	0	31	58	36
	Pmax ($\%$)	0	0	0.5	0.99	1.26	0.63	2.42	0	0	1.04	1.09	1.99
	f(Pmax) (GHz)	-	-	3.68	1.84	1.9	3.6	3.68	-	-	11.24	11.2	10.86

**Table 2:** Optimized resistances of distributed resistors in the structure with back loop and their maximum normalized absorption power at corresponding frequency.

Index		1	2	3	4	5	6	7	8	9	10	11	12
**Lateral**	Res. ($\Omega$)	57	0	130	570	771	448	0	4	243	254	288	233
	Pmax ($\%$)	0.99	0	1.01	1.18	1.5	1.44	0	0.75	2.4	1.85	1.6	2
	f(Pmax) (GHz)	13.48	-	4.48	12.64	2.32	3	-	5.22	1.8	1.84	3.48	6.14
**Side** **Cores**	Res. ($\Omega$)	0	296	933	1248	1271	979	355	21	669	864	1040	1169
	Pmax ($\%$)	0	2.09	1.78	2.02	2.06	1.85	2.73	1.89	1.26	1.04	1.38	3.21
	f(Pmax) (GHz)	-	1.7	1.76	1.8	1.82	1.86	5.46	3.62	4.8	7.76	11.14	11.06
**Center**	Res. ($\Omega$)	84	217	597	746	721	515	60	180	156	74	136	476
	Pmax ($\%$)	1.79	1.04	0.49	0.54	0.59	0.59	2.67	0.7	0.69	0.54	0.46	0.25
	f(Pmax) (GHz)	1.6	1.6	1.6	2.26	2.28	4.6	2.26	3.32	4.08	6.34	2.12	18.89
**Loop**	Res. ($\Omega$)	116											
	Pmax ($\%$)	0.69											
	f(Pmax) (GHz)	1.6											

The set of optimized resistors, as presented in Table 2, was also applied to the structure shown in Fig. 11a-ii, which differs from that of Fig. 11a-i in the arrangement of the metal wings on both sides of the substrate. The first four wings of the structure in Fig. 11a-i are arranged in T-B-B-T sequence and this structure has two wings, forming the center core, on opposite sides of the substrate. In contrast, the second structure has first four wings arranged in a T-B-T-B sequence and has two central core wings positioned on the same bottom layer. The approximation in responses of these two structures, with the same resistor values set corresponding those in Table 2, is demonstrated in Fig. 11b and 11c, thus the next analyses in this section were implemented for the structures with T-B-B-T sequence of wing metal layers.

**Fig. 12:**
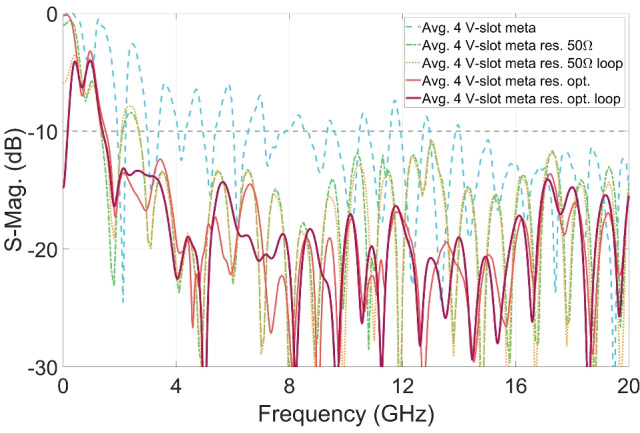
Improvement in S-parameters with different sets of distributed resistors on two structures without and with back loop.

Figure 12 presents the averaged S-parameters at the four excitation ports for five configurations: the reference 4 V-slot structure incorporating the metamaterial structure without absorption components, and four 4 V-slot structures combining the metamaterial and absorption components, without and with the back loop, and employing either $50 \ \Omega$ resistors or optimized resistors sets. While the S-parameters are already improved when all resistors are set to $50 \ \Omega$, they are significantly improved with the sets of optimized resistors as compared with the response of the reference 4 V-slot. For both structures, without and with the back loop, the S-parameters are below -10 dB across the frequency range of 1.6 to 20 GHz when optimized resistors are employed. Additionally, impedance matching is achieved at DC for structure with the back loop and optimized resistors. However, due to technology limitations, constraints in antenna size, and the number and location of the dissipation paths and resistors, the residual EM energy cannot be absorbed completely at low frequency band, leading to significant reflection between 0.16 GHz and 1.6 GHz.

**Fig. 13:**
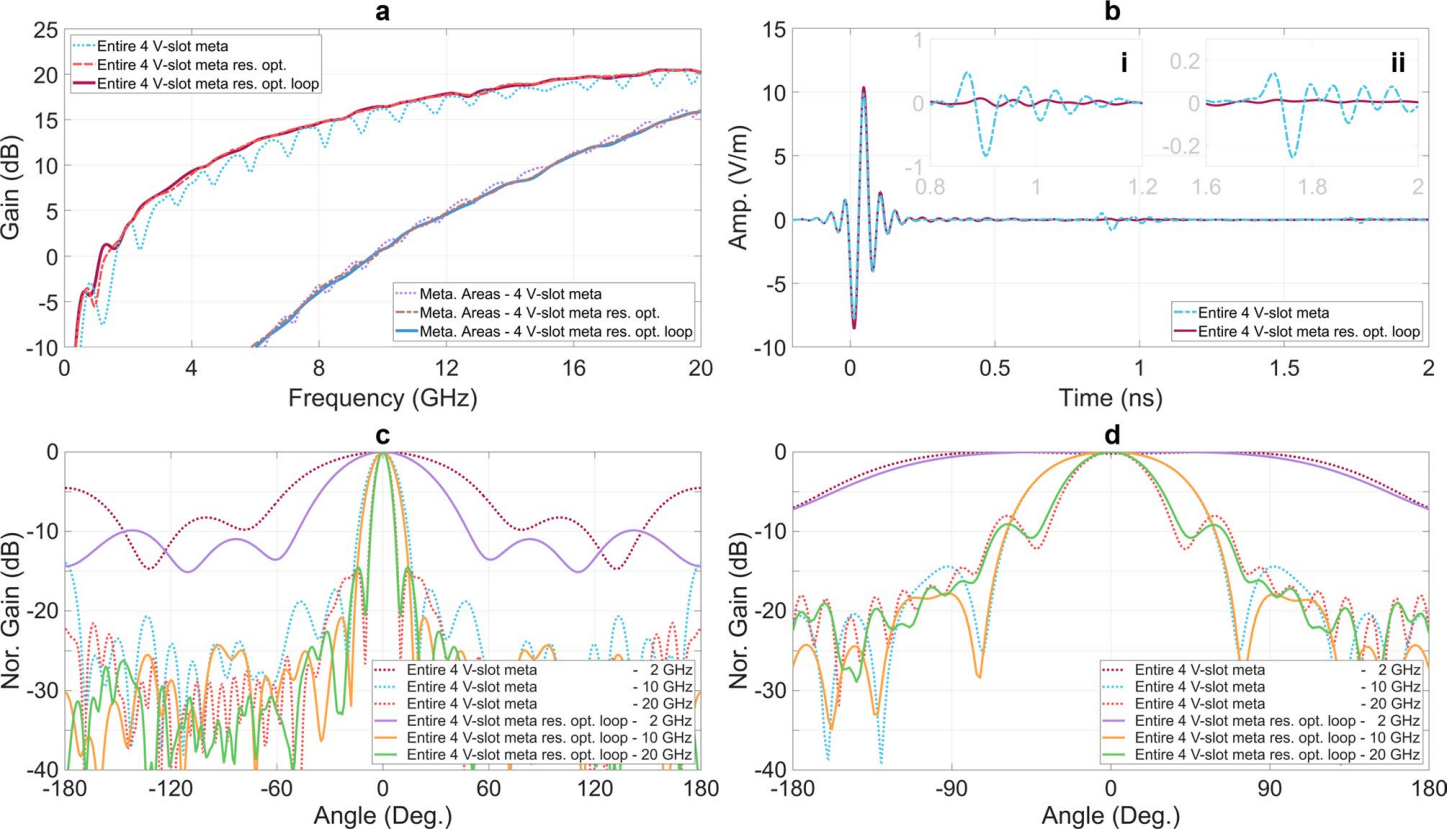
Improvement in (**a**) gain, (**b**) far-field time responses, (**c**) E-plane and (**d**) H-plane radiation pattern of structures with optimized distributed resistors.

The gains calculated for entire structure and only for the metamaterial areas are presented in Fig. 13a. This illustrates improvement gain of the two 4 V-slot structures, one without the back loop and one with the back loop, both employing the optimized sets of resistors when compared with the reference 4 V-slot with only the metamaterial. The maximum gain reach 20.5 dBi at 19 GHz and the gain curves are flattened across the entire available impedance matching band, ranging from 1.6 to 20 GHz. The gain curves of the 4 V-slot structures with optimized resistors are flattened and match all peaks of the ripple gain curves obtained for the 4 V-slot without absorption components. This indicates that the residual EM energy flows negatively influence the gain performance of the 4 V-slot without absorption components. The application of the absorption components with optimized resistors has effectively absorbed residual EM energy and mitigated these negative effects, resulting in nearly complete elimination of the gain ripples and a recovery to all gain peaks across the entire frequency band. For the structure with the back loop, a sensitivity of S11 and the gain with respect to the resistance is investigated. An assessment of a $\pm 5\%$ resistance tolerance applied to all resistors shows a maximum variation of 0.1 dB in the S11 and 0.01 dB in the gain.

The time-domain far-field response of entire structure for a Gaussian-smoothed excitation signal, as also employed in^27^, is shown in Fig. 13b. The E-field time responses were computed at a distance of 1 m from the antennas, in the main radiation direction and for the Y-polarization. This result demonstrates an improvement in antenna directivity, evidenced by an increased amplitude of the radiated signal for the structure using the set of optimized resistors. A key feature represented in Fig. 13b is the significant suppression of the repeated radiation pulses, which are caused by the residual EM flows traveling back and forth many times along the scattering paths. This is illustrated in detailed in Fig. 13b-i and 13b-ii, where the structure with optimized resistors shows a substantial reduction in the amplitude of these repeated pulses. The normalized patterns at 2, 10 and 20 GHz in both E-plane and H-plane for the 4 V-slot structure, without and with the optimized 61 resistors sets, are presented in Fig. 13c and d. In the E-plane, the main lobes of the structure with the optimized resistors are noticeably narrower compared to those without the optimized resistors, while the front-to-back ratios are higher in the case with the optimized resistors. These observations demonstrate the influence of the residual EM flows on different radiation directions and highlight the effectiveness of the resistors in suppressing undesired radiation in different directions even in the main direction, as evidenced by the reduction in the gain ripple and the elimination of the repeated radiation pulses as shown in Fig. 13a and b, respectively. On the other hand, the normalized H-plane patterns presented in Fig. 13d show insignificant differences between the both structures.

## Realization of multi V-slot and array antenna with novel full-band power dividers

In the preceding sections, multi V-slot antenna structures were examined under the assumption of ideal excitation sources. In practice, however, achieving the simultaneous and balanced excitations across all antenna ports involves the use of power dividers. These dividers must satisfy specific requirements, including full-band operation, balance and uniform port impedance, posing significant design challenges. In this section, a novel approach is introduced for the design of power dividers. This approach employs a combination of serial and parallel T-junctions to form $4^n$-port dividers. The integrations of a 4-port divider into a 4 V-slot antenna, as well as a 16-port divider into a $4 \times 4$ V-slot antenna array, are demonstrated. The responses and performances of both integrated structures are also presented and discussed.

### Novel quad-port dividers using serial and parallel T-junctions combination

**Fig. 14:**
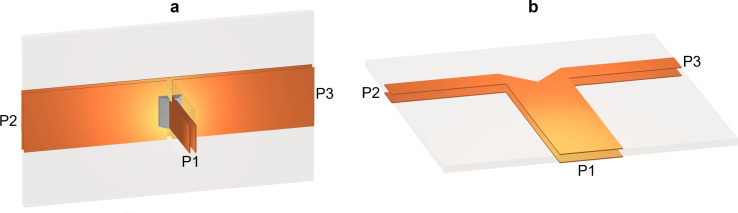
(**a**) Serial T- junction and (**b**) parallel T-junction.

Figure 14 shows the two basic elements of the proposed dividers, both designed on Roger RT5880 substrates with a thickness of 0.254 mm. As shown in Figure 14a, the serial T-junction comprises a $50 \ \Omega$ strip line connected to two $25 \ \Omega$ strip line sections serially connected at center. The input port P1 is located at the end of the $50 \ \Omega$ strip line while the two output ports are connected to the ends of the two $25 \ \Omega$ strip line sections. The input $50 \ \Omega$ stripline impedance is matched to two serially connected $25 \ \Omega$ strip line sections at the junction across the full-band. Figure 14b depicts the parallel T-junction, where a $25 \ \Omega$ strip line is connected to two $50 \ \Omega$ strip line branches, which are connected in parallel. The input signal is applied at the port P1, positioned at the end of the $25 \ \Omega$ strip line, while the two output ports P2 and P3 are located at the ends of the two $50 \ \Omega$ strip line branches. The input $25 \ \Omega$ strip line is matched to two parallel-connected $50 \ \Omega$ strip line branches at the junction, achieving matching across full-band.

**Fig. 15:**
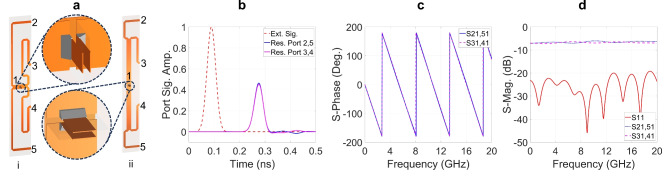
(**a**) Quad-port divider with (**a**-i) vertical and (**a**-ii) horizontal orientations of input port substrate and (**b**, **c**, **d**) the responses of horizontal input port substrate orientation divider.

Using these two basic elements, $4^n$-port power dividers can be designed, ensuring a uniform impedance of $50 \ \Omega$ at all ports. Figure 15a illustrates the two arrangements, which consist of one serial T-junction and two parallel T-junctions to form first-level ($4^1$-port) hierarchical power dividers, as shown in Fig. 15a-i and 15a-ii. The four $50 \ \Omega$ stripline output ports of these structures are built on the same substrate with adjacent ports spaced 20 mm apart. The $50 \ \Omega$ input stripline is fabricated on a different substrate, oriented either vertically as Fig. 15a-i or horizontally as Fig. 15a-ii. These orientations determine the feasibility and characteristics of second-level hierarchical dividers when multiple first-level hierarchical dividers are combined.

The main distinction between the two dividers depicted in Figures 15a-i and 15a-ii is the phase relationship of the signals at the output ports. In the structure shown in Fig. 15a-i, the signals at ports 3 and 4 are in-phase but are in antiphase with those at ports 2 and 5. Conversely, in the arrangement shown in Fig. 15a-ii, the signals at ports 2 and 3 are in phase, while being 180 degrees out of phase with those at ports 4 and 5. This phase relationship governs the arrangement of the wings in the 4 V-slot structure when the power divider is integrated into the antenna structure to achieve co-oriented and balanced E-fields along the Y-axis. Thus, the dividers illustrated in Fig. 15a-i and 15a-ii are suited for integration with the 4 V-slot antenna structures shown in Fig. 11a-i and 11a-ii, respectively. In the structure shown in Fig. 11a-i, the first four wings are sequentially fabricated on the top-bottom-bottom-top (T-B-B-T) sides of the substrate. In contrast, the antenna depicted in Fig. 11a-ii has the first four wings arranged in a top-bottom-top-bottom (T-B-T-B) sequence.

The structural simplicity and the balance in both the number of segments and the total lengths of all strip line paths from the input port to the output ports were the primary criteria to evaluate the effectiveness of the dividers. Based on these criteria, the design in Fig. 15a-ii is more favorable than that in Fig. 15a-i, and is therefore chosen for the excitation realization of the 4 V-slot antenna.

The responses of the divider shown in Fig. 15a-ii are presented in Fig. 15b, 15c and 15d, which clearly demonstrate that the input signal is distributed simultaneously and equally to all output ports across the full-band. Figure 15b shows that the pulse signals at the output ports have identical amplitudes and ToAs, while Fig. 15c and 15d indicate that the scattering parameter S21, S31, S41 and S51 are uniform in both phase and amplitude. Additionally, the impedance matching at input port is confirmed by the S11 parameter being below -20dB across the full-band.

**Fig. 16:**
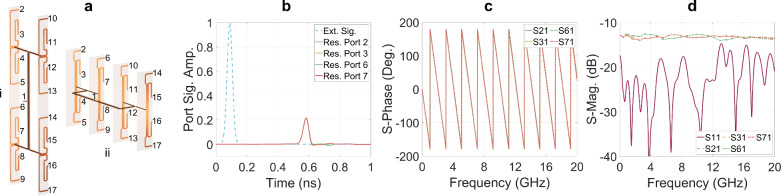
(**a**- i) $2 \times 8$-port divider, (**a**-ii) $4 \times 4$-port divider and (**b**, **c**, **d**) the responses of $4 \times 4$-port divider.

Figure 16a-i presents a $4^2$-ports power divider, which consists of four first-level hierarchical 4-port structures and one second-level hierarchical 4-port power divider. The four first-level hierarchical 4-port power dividers are evenly arranged on two substrates to drive a $2 \times 8$ V-slot antenna array. The structure depicted in Fig. 16a-ii also consists of four first-level hierarchical 4-port power dividers and one second-level hierarchical 4-port power divider. However, in this case, the four first-level hierarchical 4-port power dividers are placed on four separate substrates to drive a $4 \times 4$ V-slot antenna array.

The responses of the $4 \times 4$-port power divider shown in Fig. 16a-ii are presented in Figs. 16b, 16c and 16d. As compared to the results for a single quad-port divider shown in Figs. 15b, 15c, and 15d, the amplitudes of the signals at the output ports of the $4 \times 4$-port power divider are halved, as the number of output ports increases by a factor of four. The phases and the ToAs of the pulse signals at all output ports remains identical; but the delay of the ToA is approximately doubled when compared to the single quad-port divider due to the additional strip line lengths in the second-level hierarchical divider. However, the scattering parameter S21, S31, S61 and S71 exhibit 1dB ripples around -12.5dB, and there is an increase in S11, reaching -15dB, as shown in Fig. 16d. This is due to the increased number of discontinuities, as the number of T-junctions is doubled and one bend is added in each path from the input port to the output ports. These discontinuities generate higher-order EM energy flows and also increase the reflection back to the input port. As a result, this reduces the energy concentration and deteriorates the frequency independence of the EM energy at each output port.

### Antenna and array with integrated dividers

**Fig. 17:**
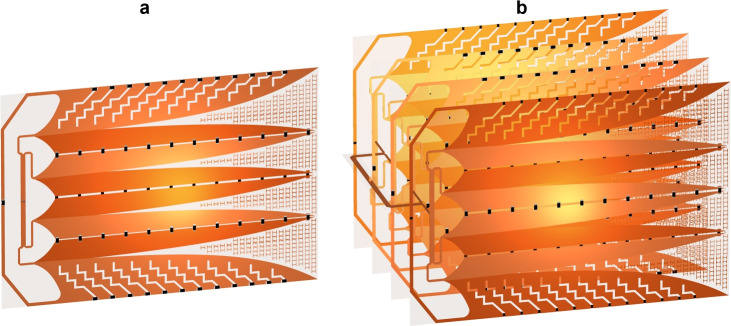
(**a**) 4 V-slot antenna integrated 4-port divider and (**b**) $4 \times 4$ V-slot array combined with $4 \times 4$-port divider.

The 4-port power divider was integrated into the 4 V-slot antenna, forming a structure with dimensions of $145 \times 100~ \text {mm}^2$, as shown in Fig. 17a. Additionally, four such 4 V-slot antennas were also combined with the $4 \times 4$-port power divider to create an array as illustrated in Fig. 17b. The spacing between adjacent antennas in the array is approximately 33.33 mm, and the total volume of the assembled array is approximately $154 \times 100 \times 100 ~ \text {mm}^3$. The mirror symmetry of the two antenna pairs in the array was arranged to fit the phase relationship at the output ports of the divider.

**Fig. 18:**
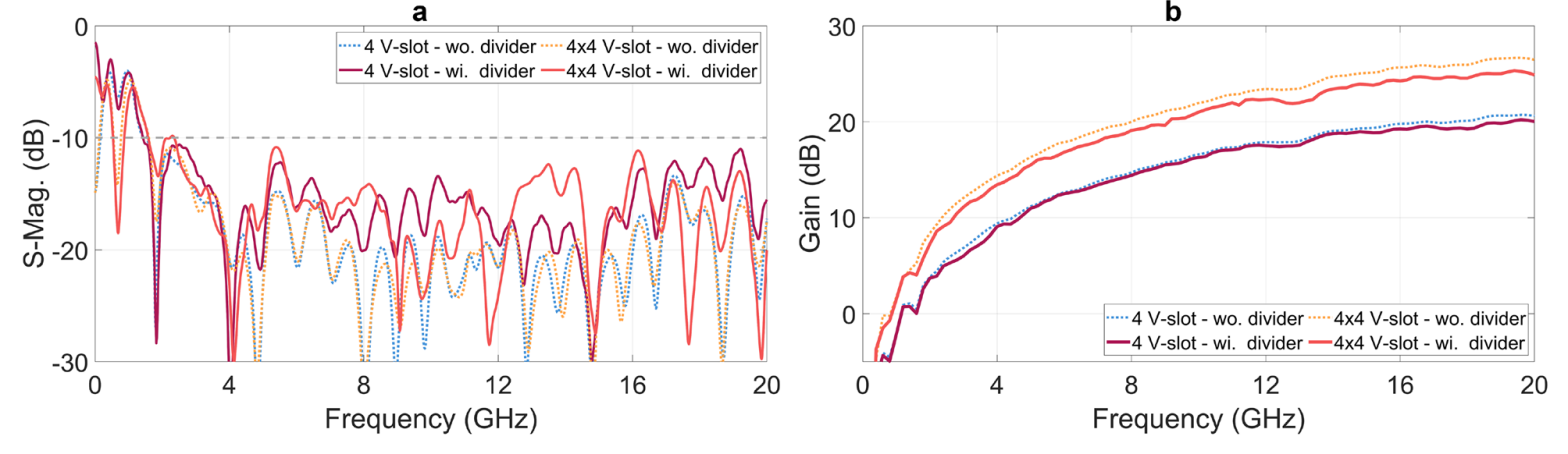
(**a**) S-parameters and (**b**) gains of 4 V-slot antenna and 4x4 V-slot array.

Figure 18 presents the responses of the 4 V-slot antenna and 4$\times$4 V-slot array, both without and with the integrated power dividers. The S-parameters shown in Fig. 18a illustrate the magnitude of average S-parameters at the all antenna excitation ports in the absence of power dividers, and the magnitude of the S-parameters at input ports of the dividers with the present of the power dividers. The results demonstrate that both the 4 V-slot antenna and the $4 \times 4$ V-slot array achieve an impedance bandwidth ranging from 1.6 to 20 GHz. However, the introduction of the dividers leads to a noticeable increase in the reflected EM energy at the input ports. This effect is more prominent in the $4 \times 4$ V-slot array with the $4 \times 4$-port power divider, where the magnitude of S11 rises to approximately -10 dB around 2.2 GHz.

The 4 V-slot antenna with the divider was integrated using the resistances listed in Table 2, while each 4 V-slot within the $4 \times 4$ V-slot array was integrated with a new set of resistances, as provided in Table 3. This new resistance set was specifically optimized for the 4 V-slot structure combined with the 4-port power divider. During the optimization process, minimizing the reflection at the input port of the divider was given higher priority, particularly for the band around 2.2 GHz, where the structure exhibited poor response. This adjustment enhances the absorption efficiency of each 4 V-slot antenna, thereby mitigating residual EM energy at this frequency. This optimization compensates for the negative impacts observed in the S-parameter of the structure, where the $4 \times 4$ V-slot array is integrated with the two-level hierarchical power divider, as shown in Fig. 18a. These negative effects arise from multiple factors, including imbalance in the responses of each 4 V-slot and array antennas, incomplete absorption of residual EM energy at each 4 V-slot, and scattering/reflection caused by the discontinuity in the two-level hierarchical divider.

**Table 3:** Resistances ($\Omega$) of distributed resistors in each 4 V-slot structure with a divider, within the $4 \times 4$ V-slot array.

Index	1	2	3	4	5	6	7	8	9	10	11	12
**Laterals**	0	0	0	665	924	568	0	0	41	48	24	181
**Side Cores**	0	41	954	1361	1450	1202	791	0	649	848	1029	1161
**Center**	99	321	704	880	849	609	0	172	157	101	181	483
**Loop**	51											

Figure 18b shows the gains of the 4 V-slot antenna and the $4 \times 4$ V-slot array, both without and with the power dividers. The 4 V-slot antenna with integrated power divider exhibits the gain ranging from 0 to 20 dBi across the frequency band from 1.6 to 20 GHz. This gain exhibits a reduction of approximately 0.5 dB at the local frequency bands of 3-4 GHz and 15-20 GHz, when compared to that of the structure without divider, where excitation is applied directly at the four antenna ports. In addition to transmission losses with the divider structure, the mismatch between the antenna and the divider also contributes to these minor dips in gain.

Quadrupling the number of antenna elements in the horizontal direction results in an array with a radiating aperture of $100 \times 100 ~ \text {mm}^2$ in both dimensions. This expansion increases the gain of the array by approximately 6 dB, reaching a maximum gain of 26 dBi at 19 GHz when the array is excited directly at all 16 ports, as shown in Fig. 18b. Although the introduction of the two-level hierarchical $4 \times 4$-port divider degrades the array gain by approximately 1 dB, the array still maintains a gain range from 5 to 25 dBi across the entire impedance band of 1.6 to 20 GHz.

**Fig. 19:**
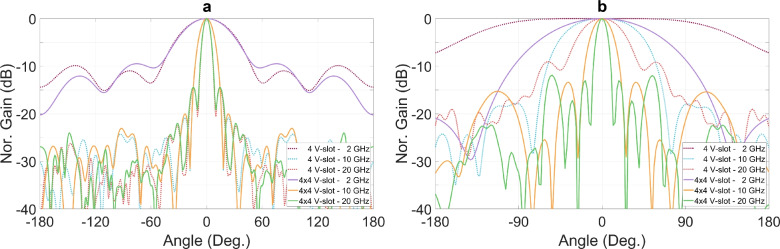
Radiation patterns of 4 V-slot antenna and 4x4 V-slot array in (**a**) E-plane and (**b**) H-plane.

Figure 19 presents the radiation pattern in E-plane and H-plane for both the 4 V-slot antenna and the array at frequencies of 2 GHz, 10 GHz and 20 GHz. Since the radiating aperture dimension in the vertical direction remains the same for both the single antenna and the array, their radiation patterns in E-plane are approximately at the same level across all three frequencies. In contrast, the increase in the dimension of the horizontal aperture of the array significantly narrows the beamwidth in the H-plane at each frequency, as compared to the single 4 V-slot antenna.

**Fig. 20:**
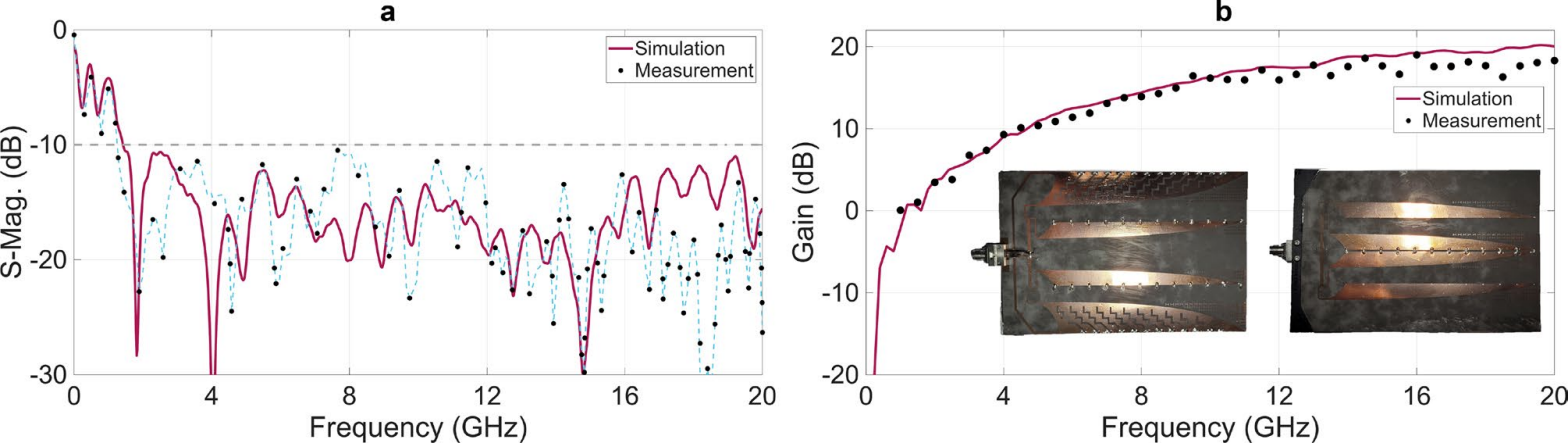
Simulated and measured (**a**) S-parameters and (**b**) gain, with fabricated prototype of 4 V-slot antenna.

Figure 20 shows the fabricated prototype and a comparison of the measured and simulated S-parameters and gain of the proposed 4 V-slot antenna. The prototype in Fig. 20(b) employs a horizontally oriented input port implemented as a $50 \ \Omega$ parallel stripline, with one end rigidly mounted and soldered perpendicular to the antenna board, while the other end is lengthened, twisted, and bent over the back loop to interface with the solderless PCB end of a 2.4 mm – PCB adapter mounted at the center of the antenna board back edge. This feeding configuration ensures mechanical robustness while minimizing radiation disturbance through backside coaxial excitation. The measurements were performed using the main instruments, including an Agilent E5071C VNA and a Rohde & Schwarz FSP spectrum analyzer. As shown in Fig. 20(a), the measured S11 demonstrates an impedance bandwidth of 1.2–20 GHz, extending toward lower frequencies relative to the simulations, while moderate deviations are observed in the higher-frequency band.

The gain results in Fig. 20(b) show that the measured gain closely follows the simulated results over the frequency range from 1 to 10 GHz, and that a peak measured gain of 19 dBi is achieved at 16 GHz, which is consistent with the simulation. These measured results further validate the effectiveness of the design methodology for the proposed antenna structure. Above 10 GHz, the measured gain progressively exhibits more pronounced ripples that increase with frequency, together with an overall degradation trend, resulting in an approximate reduction of 1.8 dB relative to the simulated gain at 20 GHz. However, these gain ripples and degradation show a fundamentally different behavior from those caused by response correlation to residual EM flows in the wings, as reported in Fig. 4, 8(b), and 13(a), and therefore cannot be attributed to such similarity effects.

The observed frequency-dependent increases in gain ripples and gain degradation, together with the deviations in the measured S11 at higher frequencies, can be explained by several key factors. These include fabrication tolerances in both the dimensions and material properties of the prototype, differences between the practical antenna structure and the simplified resistors model employed during the optimization process, and tolerances in the resistance values of the prototype. Such non-idealities lead to deviations from the intended propagation modes of EM flows, particularly under the complex spatiotemporal distributions of EM flows throughout the structure. Consequently, the optimization of the desired EM-flow patterns for both radiation and residual EM-flow absorption becomes less effective, which in turn degrades the gain along the main radiation direction and affects the S11 response at higher frequencies. A more detailed frequency-dependent examination of these effects shows that the effectiveness of the EM-flow optimization remains ensured in the lower-frequency band, where small dimensional, modeling, and material tolerances have little to no impact on the propagation modes, and their impact becomes pronounced only at higher frequencies at which the effective dimensional tolerances become comparable to the EM wavelength.

## Conclusion

In this paper, a novel approach is proposed for the classification of internal EM energy guided within the antenna structure. The method is based on the analysis of near-field and partition far-field characteristics, enabling the separation of the internal EM energy into distinct EM flows. It differentiates the desired and scattering EM flows from the residual and undesired EM flows. This classification facilitates the physical interpretation of diverse phenomena in the antenna radiation process. The proposed method is demonstrated through its application to the design of a multi V-slot antenna, where the uniform in space and excitation time arrangement for the multi V-slots facilitates the dominant source EM flows achieving the maximum in-phase impact to the main radiation lobe. Furthermore, the propagation velocity of scattering EM flows within each lane in a V-slot is precisely adjusted using inhomogeneous metamaterial structures, leading to an improvement of antenna performance.

The significant degradation in the overall responses of the antenna, such as gain ripples and port reflections, is interpreted through the concept of residual EM flows. This issue is addressed by effectively absorbing the residual EM energy, thereby restoring gain dips toward their expected peaks in the gain curve and significantly mitigating port reflections over the full operating band. The energy absorption is realized through the establishment of dissipation paths that guide the residual EM flows toward distributed resistors. The resistance values of these resistors are further optimized using different strategies to meet specific objectives in the antenna response. Additionally, novel full-band uniform port impedance dividers have been proposed to effectively excite the 4 V-slot structure and the $4 \times 4$ V-slot array. Simulation results indicate wide impedance bandwidths of 1.6 to 20 GHz, with peak gains of 20 dBi for the single antenna and 25 dBi for the array. Measurements further demonstrate that the single antenna achieves an extended bandwidth of 1.2–20 GHz and a peak gain of 19 dBi, even in the presence of fabrication and modeling tolerances that reduce the effectiveness of establishing the desired EM flows for radiation and residual EM flow absorption.

The advantages of the proposed design methodology are evidenced not only by the improved performances of the designed antennas, but more importantly by a systematic analysis of structural scattering and radiation mechanisms. This approach demonstrates strong potential for broader applications to the design of various traveling-wave antennas.

## Electronic supplementary material

Below is the link to the electronic supplementary material.

Supplementary Material 1

## Data Availability

Selected data generated or analysed during this study are included in this published article (and its Supplementary Information files).
